# Systems Approaches Evaluating the Perturbation of Xenobiotic Metabolism in Response to Cigarette Smoke Exposure in Nasal and Bronchial Tissues

**DOI:** 10.1155/2013/512086

**Published:** 2013-10-03

**Authors:** Anita R. Iskandar, Florian Martin, Marja Talikka, Walter K. Schlage, Radina Kostadinova, Carole Mathis, Julia Hoeng, Manuel C. Peitsch

**Affiliations:** Philip Morris International R&D, Philip Morris Products SA, Quai Jeanrenaud 5, 2000 Neuchâtel, Switzerland

## Abstract

Capturing the effects of exposure in a specific target organ is a major challenge in risk assessment. Exposure to cigarette smoke (CS) implicates the field of tissue injury in the lung as well as nasal and airway epithelia. Xenobiotic metabolism in particular becomes an attractive tool for chemical risk assessment because of its responsiveness against toxic compounds, including those present in CS. This study describes an efficient integration from transcriptomic data to quantitative measures, which reflect the responses against xenobiotics that are captured in a biological network model. We show here that our novel systems approach can quantify the perturbation in the network model of xenobiotic metabolism. We further show that this approach efficiently compares the perturbation upon CS exposure in bronchial and nasal epithelial cells *in vivo* samples obtained from smokers. Our observation suggests the xenobiotic responses in the bronchial and nasal epithelial cells of smokers were similar to those observed in their respective organotypic models exposed to CS. Furthermore, the results suggest that nasal tissue is a reliable surrogate to measure xenobiotic responses in bronchial tissue.

## 1. Introduction

Humans and other mammals are equipped with a sophisticated machinery to handle carcinogens and other xenobiotic compounds. In studies assessing the effects of cigarette smoke (CS) exposure, a particular interest is given to the metabolism of xenobiotics. The metabolism of xenobiotics includes oxidative reactions by phase I enzymes that convert lipophilic chemical compounds into their hydrophilic forms, followed by phase II conjugation enzymes, and finally the phase III membrane transporters [1]. The second and the last play a role in the elimination of xenobiotic metabolites [1]. The most prominent phase I enzymes are cytochrome P450s (also known as CYPs) that detoxify or activate xenobiotic compounds [1]. The phase I enzymes are also known to be responsible for the metabolism of compounds present in CS, such as nicotine, benzene, polycyclic aromatic hydrocarbons (PAHs), and tobacco-specific nitrosamines (TSNAs) [1, 2]. The induction of a specific CYP has been utilized for the identification of a specific chemical exposure (e.g., induction of CYP1 family specifies the exposure to PAHs) [1, 2]. The roles of various CYPs on the metabolism of CS toxicants have been discussed elsewhere in great detail [3–7]. The metabolization of PAHs and TSNAs can lead to the generation of carcinogenic metabolites that can interact with genomic DNA (i.e., leading to the formation of DNA adducts) [8]. Subsequently, unrepaired DNA adducts would cause gene mutations that lead to the development of cancer (carcinogenesis) [9, 10]. Furthermore, the phase II enzymes (mainly the transferases) catalyze conjugation reactions, such as glucuronidation, sulfation, methylation, and acetylation. These reactions are aimed to detoxify xenobiotic compounds [1, 5]. Moreover, the phase III enzymes refer to the active membrane transporters responsible for the translocation of xenobiotic metabolites across cellular membranes [1, 11]. The initial member of this enzyme family is the ATP-binding cassette (ABC) family of drug transporters [1]. Nonetheless, the effects of CS on the phase III response have been mainly studied in *in vitro* systems [12, 13].

The expression of CYPs in a specific tissue may suggest a tissue-specific mechanism in response to xenobiotics [14]. Although the liver is known to be the main organ responsible for the metabolism of xenobiotics, the liver is mostly processing toxicants in blood circulation, which come directly from the digestive tract [15]. Consequently, airborne toxicants that come via breathing, including CS exposure, bypass the initial liver detoxification pathway [15]. Therefore, compared to the liver, the respiratory system is exposed to a higher concentration of these toxicants [16]. Thus, the lung and respiratory tract are relevant and valuable for the risk assessment of CS toxicants. Many lung cell types, including bronchial epithelial cells, Clara cells, type II pneumocytes, and alveolar macrophages are capable in metabolizing xenobiotic compounds [14]. Normally, the levels of CYPs in the lung are expressed at trace levels, but they are induced upon CS exposure [14]. Studies have reported that bronchial tissues of smokers exhibit higher levels of CYPs (e.g., CYP1A1 and CYP1B1) as compared to nonsmokers [16–20]. Smoking cessation can reverse the induction of CYP expression upon smoking [20]. 

CS generates a field of tissue injury throughout the respiratory tract [21]. Tissue injury in the respiratory tract of healthy smokers may precede the development of CS-associated lung diseases [21]. Alteration of the genes encoding the xenobiotic metabolism enzymes has been reported to occur in a similar manner in the nasal as compared to bronchial epithelia, thus supporting the tissue injury hypothesis. For example, increases in expression of CYP1A1 and CYP1B1 were also reported in the nasal epithelium in addition to bronchial epithelium of smokers [17, 18]. Sampling of nasal epithelia by brushing/scraping is less invasive as compared to lung biopsy, thus, providing a better opportunity to screen for respiratory diseases and understand the possible mechanism associated with CS exposure [17]. Nonetheless, identifying gene expression profiles associated with xenobiotic metabolism remains challenging because the expression of genes encoding the xenobiotic enzymes is highly variable within an individual because it may change over time [15]. In this regard, we propose that our system approaches using a network model could potentially be useful to characterize the perturbation in xenobiotic metabolism upon CS exposure.

**Figure 1:**
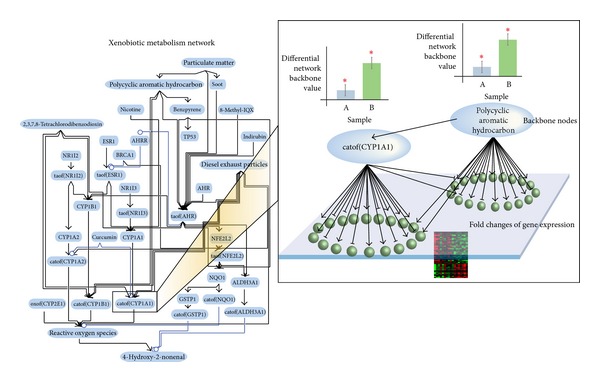
A network model representing the mechanism of xenobiotic metabolism and an illustration of network perturbation amplitude (NPA) approach.

In a qualitative manner, our network model can be used to gain insight into possible biological mechanisms pertaining to xenobiotic metabolism that are associated to a given exposure [22, 23]. The network model is built to capture biological mechanisms of the xenobiotic metabolism based on evidence from the scientific literature [24] using causal relationships encoded in Biological Expression Language (BEL) [25]. The BEL framework is an open-source technology for managing, publishing, and utilizing a structured life-science knowledge (http://www.openbel.org/) [25]. Previously, we have published the first version of the xenobiotic metabolism network model in the context of the Cellular Stress Network Model [26]. Furthermore, the network model was modified to capture a more comprehensive xenobiotic metabolism response [23] and is shown in Figure 1. The biological network model consists of backbone nodes that are connected by causal edges that carry directional information encoded in BEL. In this current xenobiotic metabolism network model, the central backbone node is the transcriptional activity of aryl hydrocarbon receptor (taof (AHR)). Aryl hydrocarbon receptor (AHR) is a transcription factor known to be activated by xenobiotic compounds. AHR regulates the expression of several target genes (e.g., CYP1A1, CYP1B1, among others). The network model uses transcriptomic data as input that are used to computationally predict the activity/functionality of the backbone nodes (Figure 1, inset) [26–29]. The blue ovals represent the activity of the backbone nodes (i.e., the functional layer) and the green balls represent the expression of genes (i.e., the transcriptional layer). The expression of a given gene can be modulated by one or more backbone nodes as depicted by black arrows. Our network model illustrates the fundamental paradigm shift from forward to backward reasoning. The former considers that the gene transcript abundance is a direct surrogate entity for its protein (or protein function). In contrast, our model considers the latter, in which the changes in gene expression are the consequence of the upstream biological processes embedded in the backbone nodes (the functional layer). Using the backward reasoning, we develop the network perturbation amplitude (NPA) algorithm that provides a quantification of the backbone nodes [23, 25, 29], which is called the “differential network backbone value” (illustrated in Figure 1, inset). Our NPA approach (described in Section 2) aims at scoring functional biological processes based on the fold changes of the gene expression. Thus, the quantification of the backbone nodes (i.e., the “differential network backbone values”) in this model reflects the biological mechanisms pertaining to xenobiotic metabolism. The NPA algorithm also integrates the network topology and directionality of edges in the network [23, 25, 29]. Both of which are taken into account for the computation of the NPA score of the entire network along with its companion statistics (described in Section 2). The NPA score of the entire network can be used to evaluate the degree of perturbation between experimental measures (e.g., exposed versus unexposed samples) [23, 29]. Thus, the differential network backbone values in this specific network exemplify the activity of biological mechanisms pertaining to xenobiotic metabolism. Moreover, using transcriptomic data derived from exposed and unexposed samples, the NPA algorithm provides a quantitative measurement of the network perturbation affecting the xenobiotic metabolism.

Our NPA approach has been used previously to compare the perturbation of the xenobiotic metabolism between an *in vivo* dataset derived from bronchial epithelium of smokers obtained by brushing and an *in vitro* dataset derived from organotypic bronchial model exposed to CS [23]. In that study, we demonstrated that at the level of backbone nodes (i.e., the differential network backbone values), the *in vivo* and *in vitro* samples were significantly correlated [23]. Here, we further extended the use of the xenobiotic metabolism network model by presenting some new use cases to probe the comparability not only between the network perturbation derived from *in vivo* and *in vitro* data but also from bronchial and its surrogate nasal tissue.

**Figure 2:**
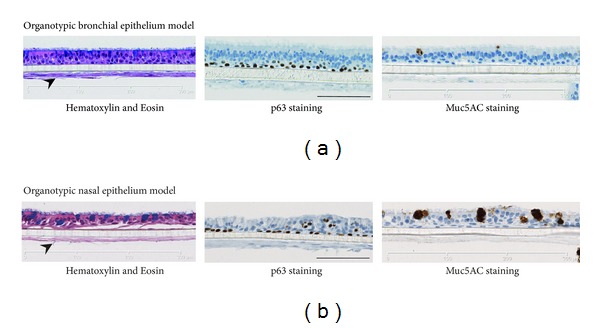
Organotypic bronchial (a) and nasal (b) models. The *in vitro* models contained ciliated cells shown in the apical layer of the Hematoxylin and Eosin stained cells (left). The models were cocultured with fibroblasts that are important for the growth and differentiation of epithelial cells (indicated by arrows). Staining of airway lineage markers: p63 and Muc5AC are shown (center and right).

We and others have recently reported that *in vitro* organotypic human-derived tracheal/bronchial epithelium pseudostratified models resemble human respiratory tract epithelium at the morphological and molecular levels [30–33]. Our group has previously reported using Gene Set Enrichment Analysis; CS exposure affects similar biological changes in bronchial epithelial cells obtained from smokers as compared to the organotypic bronchial epithelium model exposed to CS [31]. In this present study, we not only examined the *in vitro* organotypical bronchial model but also nasal model. Both the nasal and bronchial epithelia organotypic models (Figure 2) contain ciliated cells and express the airway lineage markers, such as p63—a marker of basal epithelials cells that is required for the normal development of epithelial tissues [34]—and Muc5AC that is specifically produced by airway mucous-secreting epithelial cells [35]. Specifically, in this present work, using the NPA approach and the network model, we compare the CS-induced perturbations of the xenobiotic metabolism network in: (1) nasal versus bronchial tissues *in vivo*, (2) nasal versus bronchial tissues *in vitro*, (3) nasal and bronchial tissues *in vivo* versus *in vitro*. 

## 2. Materials and Methods

### 2.1. Organotypic Tissue Culture Models

MucilAir-human fibroblasts-bronchial and MucilAir-human fibroblast-nasal full-thickness tissue models were generated from primary human respiratory epithelial cells cocultured with primary human airway fibroblasts. The MucilAir models were purchased from Epithelix Sárl (Geneva, Switzerland) and maintained according to the manufacturer's protocol. MucilAir model is a ready-to-use 3D model of differentiated human epithelium [36]. The organotypic tissues were primary human epithelial cells isolated from healthy, nonsmoking, Caucasian donors that were reconstituted using fibroblasts. Coculture of fibroblasts has been shown to contribute to the growth and differentiation of epithelial cells in 3D cultures [37]. The bronchial epithelial cells were obtained from one particular donor, and the nasal epithelial cells were obtained from another donor. Quality control assessments were performed on both models (data not shown). The tissue models were cultured at the air-liquid interface in 0.7 mL media in cell culture inserts (24-well format). The organotypic models were maintained at 37°C for 14 days at the air-liquid interface with fresh medium replaced every 2 days. 

### 2.2. Vitrocell Cigarette Smoke Exposure to the Respiratory Organotypic Tissue Culture Models

After cell culture models grown in culture for 2-3 days, the tissues (in triplicate) were exposed at the air-liquid interface to 16% (vol/vol) mainstream CS exposure (a total of 4 cigarettes, 3R4F) with 1 hour rest between each cigarette and 60% humidified air in the Vitrocell systems (Waldkirch, Germany). The 60% humidified air exposure was used as a control exposure. The Total Particulate Matter (TPM) inside the exposure chamber has been measured for each CS concentration (the mean TPM deposition measured after each cigarette was 2842.4 ng/cm^2^ ± SEM = 570.7, *N* = 24). The reference cigarette 3R4F was obtained from the University of Kentucky (http://www2.ca.uky.edu/refcig/) and smoked on the 30-port carousel smoking machine (SM2000, Philip Morris, Int.) according to the Health Canada regimen [38]. After exposure, the organotypic models were incubated with fresh culture medium immediately (0 h after exposure). Additionally various durations of postexposure were implemented (4, 24, and 48 h) before tissues were harvested for further analyses. 

### 2.3. RNA and Microarray Hybridization

Exposed tissues (*n* = 3) at 0, 4, 24, and 48 h postexposure time were washed 3 times with ice-cold PBS and subsequently lysed using Qiazol lysis reagent (miRNeasy Mini Kit, Qiagen) and frozen at −80°C for up to 1 week. The miRNeasy Mini Kit was used for the extraction and purification of mRNA. Total RNA quantity was measured using NanoDrop ND1000 and qualitatively verified using an Agilent 2100 Bioanalyzer profile (A RIN number greater than 8). For the mRNA analysis, total RNA (100 ng) was processed according to the GeneChip HT 3′ IVT Express User Manual (Affymetrix). Genechip Human Genome U133 Plus 2 Arrays were used for microarray hybridization. The dataset has been submitted to Arrayexpress (Accession code = E-MTAB-1721).

### 2.4. Microarray Data Processing

Data processing and scoring methods were implemented using the *R* statistical environment version 2.14 [39]. Raw RNA expression data were analyzed using the *affy* and *limma* packages of the Bioconductor suite of microarray analysis tools (version 2.9) available in the *R* statistical environment [40, 41]. Robust Multichip Average (GCRMA) background correction and quantile normalization were used to generate probe set expression values [42]. For each data set, an overall linear model was fitted to the data for the specific contrasts of interest (e.g., the comparisons of “treated” and “control” conditions) generating raw *P* values for each probe set on the microarray, which were further adjusted using the Benjamini-Hochberg procedure. A blocking factor (the exposure plate) from the experiment design was accounted in the model for data processing.

### 2.5. Network-Based Analysis

Leveraging the “cause-and-effect” network models together with Network Perturbation Amplitude (NPA) algorithms ([29, 43]), the fold changes of gene expression were translated into differential network backbone value for each backbone node in the network (Figure 1). The “differential network backbone value” was the result of a fitting procedure between the network model and the gene expression fold changes, where the smoothest function (accounting for the sign of the causal edges) was derived by further imposing a boundary condition on the backbone nodes corresponding to the gene expression changes. Statistical correlations were computed for the differential network backbone values and fold changes of gene expression, including *R* ^2^, Pearson correlation, and Spearman correlation, along with the *P* values. For a negative control analysis, we computed a permutation test to assess if the correlation obtained between the differential network backbone values were solely due to the dimension reduction effect. Genes underlying the network were randomly permuted 1000 times for each comparison group to decorrelate the fold changes of gene expression (the GSE16008 nasal versus bronchial data were used in this example). Subsequently, correlations between the backbone values were computed. This approach leads to a *P* value 0.002 two sided (see Supplementary Figure 1 in the Supplementary Material available online at http://dx.doi.org/10.1155/2013/512086).

The differential network backbone values were in turn summarized into a quantitative measure of NPA score for the entire network. The NPA is computed as a (semi-)Sobolev-type norm on the signed directed graph underlying the network (*N*), (which can be expressed as a quadratic form). In summary, the NPA algorithm considers two main input components. First the “cause-and-effect” network model describing the mechanism and second, the gene expression dataset from a well-designed experiment. 

In addition to the confidence intervals of the NPA scores, which account for the experimental error (e.g., the biological variation between samples in an experimental group), companion statistics were derived to describe the specificity of the NPA score to the biology described in the network. Because NPA is a quadratic form of the fold changes, its variance can be computed based on the fold-change estimated variances. A confidence interval is subsequently derived using the central limit theorem. Two permutation tests were implemented [43], whereby first, to assesses if the results were specific to the underlying evidence (i.e., gene fold-changes) in the model, leading to a permutation *P* value (denoted by *O in the figures when *P*-value < 0.05). Second, to assess whether the “cause-and-effect” layer of the network significantly contributed to the amplitude of the network perturbation (denoted by K* in the figures when *P* value < 0.05). The network was considered to be specifically perturbed if both *P* values mentioned before were <0.05, and the perturbation was called significant when the confidence interval was greater than 0.

### 2.6. Cytochrome Activity Assay

We measured the activity of CYP1A1 and CYP1B1 using nonlytic P450-Glo assays (CYP1A1 assay cat number V8752; CYP1B1 assay cat number V8762; Promega) based on luminescence at the 48 h after exposure on the human organotypic nasal and bronchial models. The assay was performed according to the manufacturer's recommendations. Briefly, both nasal and bronchial epithelia models were incubated in medium with luminogenic CYP-Glo substrate, such as luciferin-CEE for 3 h (CYP1A1 and CYP1B1), to produce a luciferin product that can be quantified in the supernatant by a light-generating reaction upon the addition of luciferin detection reagent.

## 3. Results and Discussion

### 3.1. Comparison between Xenobiotic Metabolism Responses in Bronchial and Nasal Epithelia *In Vivo* upon CS Exposure

Another group has reported that alterations of xenobiotic metabolism in the bronchial epithelium obtained from human donors are similar to those in the nasal epithelium [21]. This observation supports the field of tissue injury hypothesis, in which changes in the respiratory tract of smokers precede the development of CS-associated lung diseases [21]. To further examine this hypothesis, we used the NPA approach and the xenobiotic network model to compare the differential network backbone values derived from the bronchial and nasal samples in the *in vivo* dataset GSE16008 (smokers versus nonsmokers). This approach was taken to compare the biological mechanisms associated to xenobiotic metabolism that were perturbed by CS exposure in these two tissues. 

**Figure 3:**
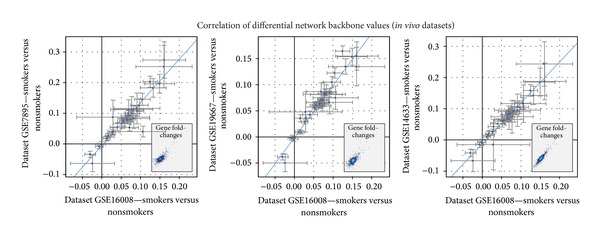
Comparability of GSE16008 dataset to other publicly available datasets. Correlations among the differential network backbone values from different human datasets in the xenobiotic metabolism network were shown. The human datasets comprise smoker versus nonsmoker data. Each data point represents a backbone node in the network. The 95%-confidence intervals of the differential network backbone values are shown for the two perturbations (axes). Blue lines show the linear regression lines computed by least squares fit. All the regression models were significant (*P* value < 0.05). Insets illustrate the correlation of the fold change of gene expressions.

We have reported before that the xenobiotic metabolism network model can capture the common perturbation from several independent datasets [22, 23]. In this study, the publicly available dataset (GSE16008) was used to compare the xenobiotic responses in the human bronchial and nasal epithelia upon CS exposure. The GSE16008 dataset contains gene expression from nasal and bronchial epithelial cells obtained from healthy current smokers and nonsmokers. The bronchial epithelial cells were collected by bronchoscopy, whereas the nasal epithelial cells were collected by brushing the inferior turbinate [18]. Because we have not analyzed GSE16008 before, we first probed the comparability of this particular dataset to other publicly available datasets (i.e., GSE7895, GSE19667, and GSE14633). These datasets contain gene expression of bronchial epithelial cells obtained by bronchoscopy from smokers and nonsmokers (GSE7895, [20]), (GSE19667, [44]), and (GSE14633, [45]).  Figure 3 shows the correlation of the differential network backbone values in the xenobiotic metabolism network model (the model is depicted in Figure 1) using the NPA approach between GSE16008 and the aforementioned datasets.

**Figure 4:**
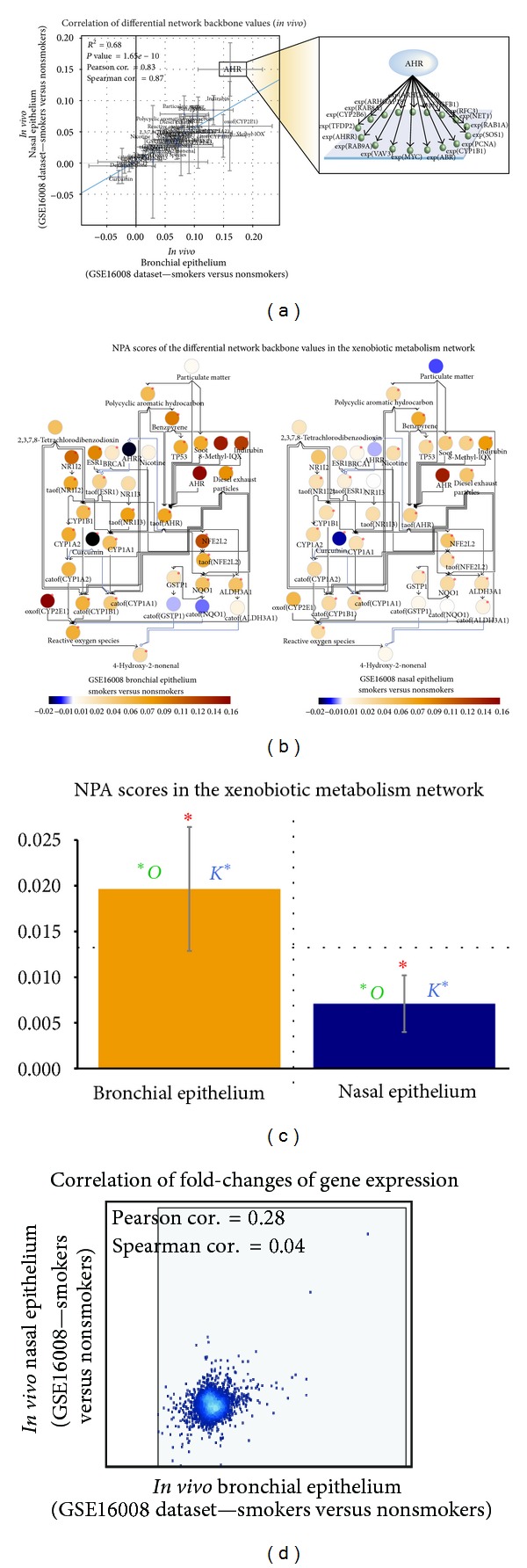
Correlation between the differential network backbone values in response to CS exposure generated from *in vivo* human bronchial and nasal datasets in the xenobiotic metabolism network model.

Figure 4(a) shows that the differential network backbone values were well correlated between the *in vivo* bronchial and nasal brushing epithelia. Furthermore, we illustrated how the backbone AHR was computed from the gene expression data (Figure 4(a), inset). Figure 4(a) shows a correlation of the differential network backbone values derived from the bronchial and nasal samples in the *in vivo* dataset GSE16008 (smokers versus nonsmokers) computed using the NPA approach in the xenobiotic metabolism network model. Each data point represents a backbone node in the xenobiotic network model. Representative node labels are shown. The blue line is the linear regression line computed by least squares fit with significant *P* value < 0.05. The 95%-confidence intervals of the differential backbone values are shown for the two perturbations (axes). The differential network backbone values generated from the *in vivo* bronchial data were in general greater than those from the *in vivo* nasal data (inferred from the regression line), with the exception of aryl hydrocarbon receptor (AHR) (Figure 4(a)). 

Figure 4(b) captures the differential network backbone values in the xenobiotic metabolism network model using the *in vivo* bronchial (left) and nasal (right) data. The different colors reflect the quantification of the backbone nodes (i.e., the “differential network backbone values”) derived from the NPA algorithm that demonstrate the biological mechanism pertaining to xenobiotic metabolism. The values less than 0 indicate downregulation of the backbone node activity; whereas, the values more than 0 indicate upregulation of the backbone node activity. *P values < 0.05. Furthermore, the nature of the network perturbation, which was reflected on the differential network backbone values, was similar between the bronchial and nasal epithelia. For example, cigarette smoking was associated with decreased activation of the aryl hydrocarbon receptor repressor (AHRR) in both the bronchial and nasal samples (Figure 4(b)). AHRR is known to inhibit the binding of AHR to xenobiotic-responsive elements (XRE), thus, suppressing the transcription of AHR-dependent genes, including CYP1A1, CYP1A2, and CYP1B1 [46]. Consistently, we observed the upregulated differential network backbone values for these aforementioned CYPs (Figure 4(b)). Despite the expression of AHR falls farther away from the regression line (Figure 4(a) as mentioned before); interestingly, the differential network backbone values of the AHR node were similar between the bronchial and nasal, indicating that the activity of AHR upon CS exposure was increased to the same extend in both bronchial and nasal (Figures 4(a) and 4(b)). This result supports the notion that AHR plays an important role in the activation of CS toxicants not only in the lower respiratory tract but also in the upper respiratory tract. Studies have indicated that AHR is a promiscuous receptor that is capable in binding to diverse chemicals, leading to their activations [1]. In regard to smoking, this observation further supports that CS exposure generates a field of tissue injury throughout the respiratory tract [21].

Figure 4(c) shows the bar plot of the NPA scores for the entire networks along with their companion statistics along with their companion statistics *O and K* as described in Section 2 (*P* values < 0.05). These significant statistics suggest that both *in vivo* nasal and bronchial samples from the dataset significantly demonstrate the biological mechanisms represented in the xenobiotic metabolism network model (described in Section 2). These results suggest that the nasal as a surrogate tissue of the bronchial epithelium elicits similar xenobiotic responses upon CS exposure, which were reflected by the similar changes of the differential network backbone values in the xenobiotic metabolism network model. This result also supports the overall CS exposure-related impact on the tissues lining the respiratory tract [21].

Moreover, unlike the correlation at the backbone levels (i.e., functional layer) (Figure 4(a)), a correlation between the gene expression generated from the dataset GSE16008 (i.e., transcriptional layer) was not observed (Figure 4(d)). These results indicate that the utilization of our NPA approach using the network model, which comprised these two layers (i.e., functional and transcriptional layers), could facilitate a high-resolution comparison of high-throughput transcriptomic data and to understand the biological insight ingrained in the data.

### 3.2. Comparison between Xenobiotic Metabolism Responses in Organotypic Bronchial and Nasal *In Vitro* Models upon CS Exposure

**Figure 5:**
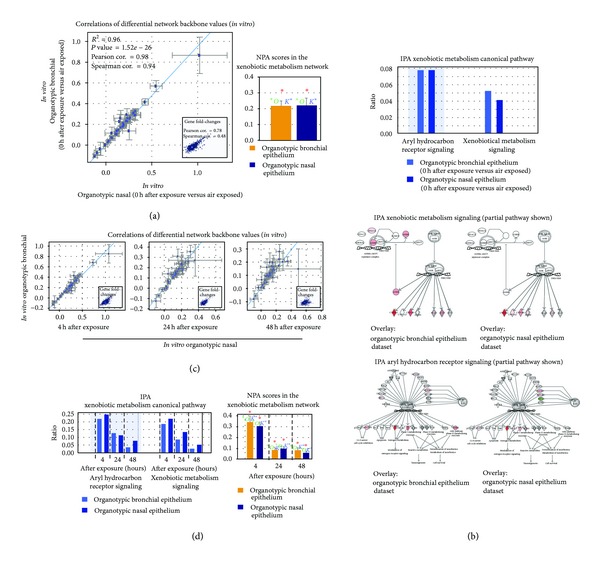
Comparison between xenobiotic metabolism responses in organotypic bronchial and nasal epithelia *in vitro* models upon CS exposure.

We further determined whether the same information could be observed *in vitro*. Development of a reliable *in vitro* system that mimics the *in vivo* condition has been challenging. Recently, organotypic culture models of human cells have been developed and utilized to understand biological processes [30–33, 47]. In this present study, we compared the network perturbations that occurred in *in vitro* organotypic bronchial and nasal epithelia models that were exposed to whole CS (see Section 2). The gene expression from these tissue models was measured in cells that were immediately harvested after the last exposure (0 h after exposure).  Figure 5(a) shows that the differential network backbone values were well correlated between the *in vitro* bronchial and nasal epithelia. This comparability at the functional layer was in agreement with what was observed using the data generated from the *in vivo* dataset. Each data point represents a backbone node in the xenobiotic network model. The blue line is the linear regression line computed by least squares fit with significant *P* value < 0.05. The 95%-confidence intervals of the differential backbone values are shown for the two perturbations (axes). Figure 5(a), inset illustrates the correlation between the fold changes of the gene expression (correlation at the transcriptional layer). The NPA scores (bar plot) indicating the statistical significance of the perturbation of the xenobiotic metabolism network model in response to smoking are shown: *indicates significance of NPA scores of the entire network level generated from the *in vitro* bronchial and nasal datasets as well as their companion statistics *O and K* as described in Section 2 (*P* values < 0.05). The bar plot (Figure 5(a)) shows the NPA scores for the entire networks along with their companion statistics. These significant statistics suggest that both *in vitro* nasal and bronchial samples from the dataset significantly demonstrate the biological mechanisms represented in the xenobiotic metabolism network model (described in Section 2).

Furthermore, to investigate how our analysis using the xenobiotic metabolism network model was comparable to the commercially available data analysis and interpretation tool (Ingenuity Pathways Analysis (IPA)), the same datasets generated from the *in vitro* organotypic samples were uploaded to IPA. Within the IPA's knowledge base, the “Xenobiotic Metabolism” canonical pathways are comprised of two signaling pathways: the “Aryl Hydrocarbon Receptor Signaling” and the “Xenobiotic Metabolism Signaling.” Figure 5(b) shows significant associations between the datasets and the two IPA's canonical pathways within the category of “Xenobiotic Metabolism” (*P* value < 0.05). The *y*-axis displays the ratio calculated as follows: the number of genes in the associated pathways that meet cutoff criteria, divided by the total number of genes that make up that specific pathway. The taller the bars, the more genes were associated with the pathway. Representative pathways overlayed with the two datasets generated from the organotypic bronchial and nasal models are shown. Interestingly, the bronchial and nasal data were associated to the two signaling pathways in a similar manner, which was indicated by the similarity of the ratios (Figure 5(b), top). This observation was in agreement with our approach using the NPA analyses and the network model (Figure 5(a)). 

Additionally, we examined the effects of various postexposure time points to assess the ability of cells to recover from CS exposure. We hypothesized that the longer the duration of postexposure, the less perturbed the xenobiotic metabolism would be. The differential network backbone values continue to be correlated between the bronchial and nasal at the 4, 24, and 48 h after exposure time (Figure 5(c)). Nevertheless, the correlations were reduced as the duration of the postexposure increased (Figure 5(c) and Table 1). Each data point represents a backbone node in the xenobiotic network model as indicated with the node labels. The blue line is the linear regression line computed by least squares fit with significant *P* value < 0.05. The 95%-confidence intervals of the differential backbone values are shown for the two perturbations (axes).  Figure 5(c), inset, illustrates the correlation between the fold changes of the gene expression (correlation at the transcriptional layer, Table 1). The reduced responses that were inferred from the reduced correlations between the differential network backbone values, were also reflected from the analysis using IPA, in which decreased ratios (i.e., the association between the datasets with the two pathways) were observed in the datasets at the later time point of postexposure (Figure 5(d), left bar plot). The results from IPA analysis were also in agreement to those reflected by the NPA scores for the entire network (Figure 5(d), right bar plot), in which the later time point of postexposure had reduced scores. Taken together, this data suggests that the shorter the postexposure time, the more perturbed the xenobiotic metabolism in both bronchial and nasal tissues. This observation is consistent with a previous study in which a transient induction of phase I xenobiotic metabolism enzymes (e.g., *cyp1A1* and *aldh3A1*) is observed in CS-exposed lung tissues of Sprague-Dawley rats [48]. Furthermore, this could offer a likely explanation for why we observed a better correlation of the differential network backbone values between the *in vitro* organotypic bronchial and nasal models with shorter postexposure time (Figure 5(c) and Table 1).

### 3.3. *In Vivo* and *In Vitro* Comparison between Xenobiotic Metabolism Responses in Bronchial and Nasal Epithelia upon CS Exposure

**Figure 6:**
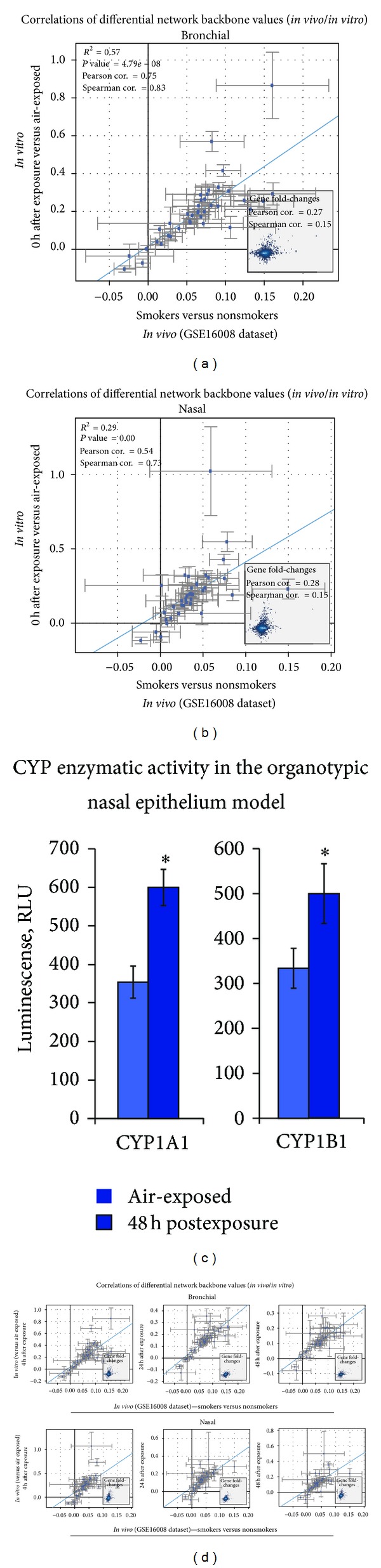
Correlations between the differential network backbone values in response to CS exposure generated from *in vivo* datasets and *in vitro* organotypic models in the xenobiotic metabolism network model.

We further examined whether *in vitro* organotypic models could reveal a similar xenobiotic response upon CS exposure as compared to that observed *in vivo*. Thus, we determined whether the differential network backbone values generated from the *in vivo* datasets were correlated to those from the *in vivo*. The NPA approach that quantifies the changes at the backbone levels (i.e., the differential network backbone values) could indicate the potential biological mechanisms that were perturbed upon exposure to CS. Therefore, whether similar biological responses occurred in *in vivo* situation were comparable to those in *in vitro* models can be inferred from the correlation between the differential network backbone values. Figures 6(a) and 6(b) show the correlations, in bronchial and nasal samples, respectively, between the differential network backbone values generated from *in vivo* dataset to those generated from *in vitro* models. This observation is in agreement with our other publication, in which a similar biological alteration was observed in *in vivo* bronchial epithelial cells as compared to an *in vitro* organotypic bronchial epithelial model (EpiAirway system, MatTeK Corporation) [31]. Nonetheless, this present study further suggests that the *in vitro* organotypic nasal model would also be useful to investigate the mechanisms occur in the *in vivo* nasal situation upon smoking. 

Moreover, we compared the data derived from organotypic *in vitro* models at various postexposure time to the *in vivo* datasets at the backbone nodes level in the xenobiotic metabolism network model; the xenobiotic responses were better correlated (Table 2) in the bronchial (Figure 6(a)) as compared to the nasal samples (Figure 6(b)). The differential network backbone values were derived from the bronchial and nasal data in the *in vivo* dataset GSE16008 (smokers versus nonsmokers) and from the data generated from CS-exposed *in vitro* organotypic bronchial and nasal models computed using the NPA approach in the xenobiotic metabolism network model. Each data point represents a backbone node in the xenobiotic network model. The blue line is the linear regression line computed by least squares fit with significant *P* value < 0.05. The 95%-confidence intervals of the differential backbone values are shown for the two perturbations (axes). The insets illustrate the correlations between the fold-changes of the gene expression (correlation at the transcriptional layer). Zhang and colleagues have previously reported that the effect of smoking is less pronounced in the nasal epithelium when compared to bronchial epithelium obtained from smokers [18], which could explain why the correlation observed in the nasal samples was weaker. Moreover, to better assess the *in vitro* organotypic nasal model, we tested the effects of CS exposure on the enzymatic activity of CYP1A1 and CYP1B1. We found that CS exposure significantly increased the activity of both CYP1A1 and CYP1B1 measured in the nasal epithelium *in vitro* model (Figure 6(c)), supporting the potential of the nasal model to be utilized for toxicity assessment against airborne exposure. The CYP activities (luminescence, RLU) of the CS-exposed nasal tissues as compared to the air-exposed tissues were measured at 48 h after exposure (see Section 2). Shown are CYP1A1 and CYP1B1 activities obtained from triplicate measurements (*N* = 3), *P < 0.05 as compared to the air-exposed tissue. Additionally, although the xenobiotic responses generated from the *in vitro* organotypic models at the later time of postexposure were reduced (Figure 5(d) and Table 2), the differential network backbone values remained well correlated as compared to those generated from the *in vivo* datasets (Figure 6(d) and Table 2). However, the *in vivo*/*in vitro* correlations of the gene expression (Figure 6(d), insets and Table 2) were weak.

## 4. Conclusion

Here we show that our quantitative systems-level approach utilizing the xenobiotic metabolism network model allowed a robust comparison derived from transcriptional data. This approach could provide a mechanistic insight that occurred in response to CS exposure, which is reflected from the differential network backbone values. The quantification of the xenobiotic network model perturbation using the NPA approach not only could compare the responses observed from datasets generated from *in vivo* samples and *in vitro* organotypic models but also from bronchial and its surrogate nasal epithelia. Furthermore, our results suggested that the organotypic nasal *in vitro* model could be useful as a risk assessment tool in understanding biological mechanisms leading to lung diseases associated to airborne exposure. Our results are consistent with an overall CS exposure-related impact on the tissues lining the respiratory tract, and thus supporting the field of tissue injury theory [21].

Studies have reported that CS exposure is associated with increased expression of genes encoding the xenobiotic metabolism enzymes, such as CYP1A1 and CYP1B1 in both the nasal [17, 18] and buccal epithelia [49, 50]. Similar to the nasal epithelium, buccal epithelium has been postulated as a suitable surrogate tissue for the lung, which could be useful to determine disease risk biomarkers [51]. Because collections of both nasal and buccal epithelial samples are relatively simpler and less invasive as compared to the collection of bronchial epithelium, these tissues become attractive surrogate tissues for toxicology assessment in response to CS exposure. Sampling of the lung is usually done by brushing or biopsy [52]. However, these methods are invasive, thus, unfeasible for large clinical studies [51]. Additionally, the use of *in vitro* organotypic models provides an attractive tool for toxicology assessments of specific airborne exposures. In this study, we also demonstrated that the perturbation of the xenobiotic metabolism in the CS-exposed organotypic nasal *in vitro* epithelia models resembled that in the nasal epithelial cells obtained by brushing from smoker donors. Nonetheless, whether similar results would be observed in organotypic model derived from different donors is unknown. Donor-dependent variability is expected [36] and should be addressed in future studies. Furthermore, future studies should also investigate whether other bronchial surrogate tissues (e.g., buccal epithelial *in vivo* samples and organotypic *in vitro* models) could be utilized to assess and compare the perturbation in the xenobiotic metabolism upon CS exposure. Such data would further highlight the relevance and practicality of *in vitro* organotypic models for toxicology assessment. 

**Figure 7:**
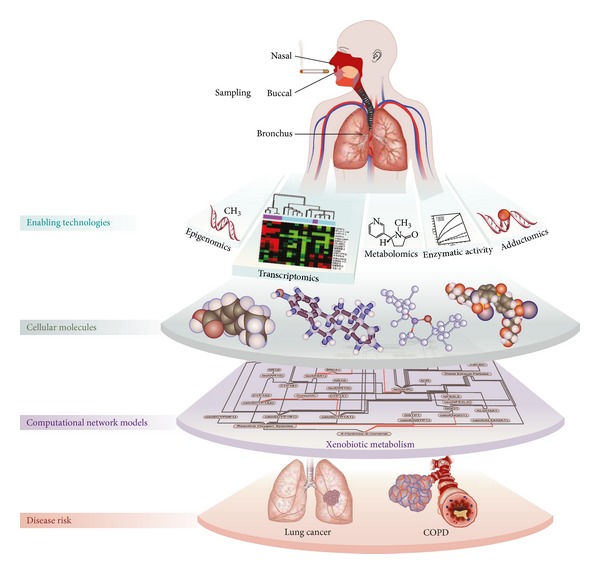
Our present work could be implemented to the new generation of “omics” technology for the overall assessment of CS exposure pertaining to the perturbation of xenobiotic metabolism. This current work provided an useful example for the utilization of transcriptomic data for impact assessment that focuses on xenobiotic responses against airborne exposure.

Our present work provided a useful example for the utilization of transcriptomic data for impact assessment that focuses on xenobiotic responses against airborne exposure. However, theories have been developed supporting how the entire respiratory tract exhibits genomic, epigenomic (e.g., methylation of genes encoding the xenobiotic metabolizing enzymes), transcriptomic, and proteomic modifications [17, 53, 54]. Additionally, CS exposure has often been associated with adduct formation not only in the lung tissue but also in the blood circulation [55–62].  Figure 7 depicts how transcriptomic data could be leveraged into various systems approaches that implement the larger spectrum of “omics” technologies. Although this present study described the utilization of transcriptomic data, further information from genome and its derivatives, including proteins, metabolites, and adducts would be useful for the overall assessment of CS exposure on the metabolism of xenobiotic. 

## Supplementary Material

The negative control analysis was generated by computing a permutation test. The permutation test is used to examine whether the correlation obtained by comparing the differential network backbone values were merely due to the dimension reduction effect. The genes underlying the network (i.e., the transcriptional layer) were randomly permuted 1000 times for each of the comparison group to de-correlate the fold-changes of gene expression (the GSE16008 nasal vs. bronchial data were used in this example). Subsequently, correlations between the differential network backbone values were computed. This approach leads to a P-value 0.002 two-sided (Supplementary Figure 1).

## Figures and Tables

**Table 1:** Statistical correlation between the bronchial versus nasal *in vitro* data.

	Between the backbone values		Between the fold change of genes expression	
Comparison group	Pearson correlation	Spearman correlation	Pearson correlation	Spearman correlation
Bronchial *in vitro* versus nasal *in vitro* (4 h after exposure)	0.97	0.95	0.72	0.55
Bronchial *in vitro* versus nasal *in vitro* (24 h after exposure)	0.93	0.94	0.62	0.49
Bronchial *in vitro* versus nasal *in vitro* (48 h after exposure)	0.77	0.86	0.39	0.37

*P* values < 0.05 for all comparisons.

**Table 2:** Statistical correlation between the *in vivo* versus *in vitro* data at various postexposure times.

	Between the backbone values		Between the fold change of genes expression	
Comparison group	Pearson correlation	Spearman correlation	Pearson Correlation	Spearman Correlation
Bronchial				
*In vivo* versus 4 h after exposure *in vitro *	0.73	0.77	0.25	0.13
*In vivo* versus 24 h after exposure *in vitro *	0.81	0.83	0.37	0.29
*In vivo* versus 48 h after exposure *in vitro *	0.77	0.80	0.35	0.30

Nasal				
*In vivo* versus 4 h after exposure *in vitro *	0.57	0.76	0.35	0.27
*In vivo* versus 24 h after exposure* in vitro *	0.71	0.74	0.31	0.09
*In vivo* versus 48 h after exposure *in vitro *	0.65	0.73	0.26	0.14

*P* values < 0.05 for all comparisons.
