# Supplementary material for: Systems Approaches Evaluating the Perturbation of Xenobiotic Metabolism in Response to Cigarette Smoke Exposure in Nasal and Bronchial Tissues
Source: Biomed Res Int. 2013 Oct 3;2013:512086. doi: 10.1155/2013/512086 (PMC3808713; doi:10.1155/2013/512086)
Supplement: Supplementary file 1 — The negative control analysis was generated by computing a permutation test. The permutation test is used to examine whether the correlation obtained by comparing the differential network backbone values were merely due to the dimension reduction effect. The genes underlying the network (i.e., the transcriptional layer) were randomly permuted 1000 times for each of the comparison group to de-correlate the fold-changes of gene expression (the GSE16008 nasal vs. bronchial data were used in this example). Subsequently, correlations between the differential network backbone values were computed. This approach leads to a P-value 0.002 two-sided (Supplementary Figure 1). [file 512086.f1.pdf]

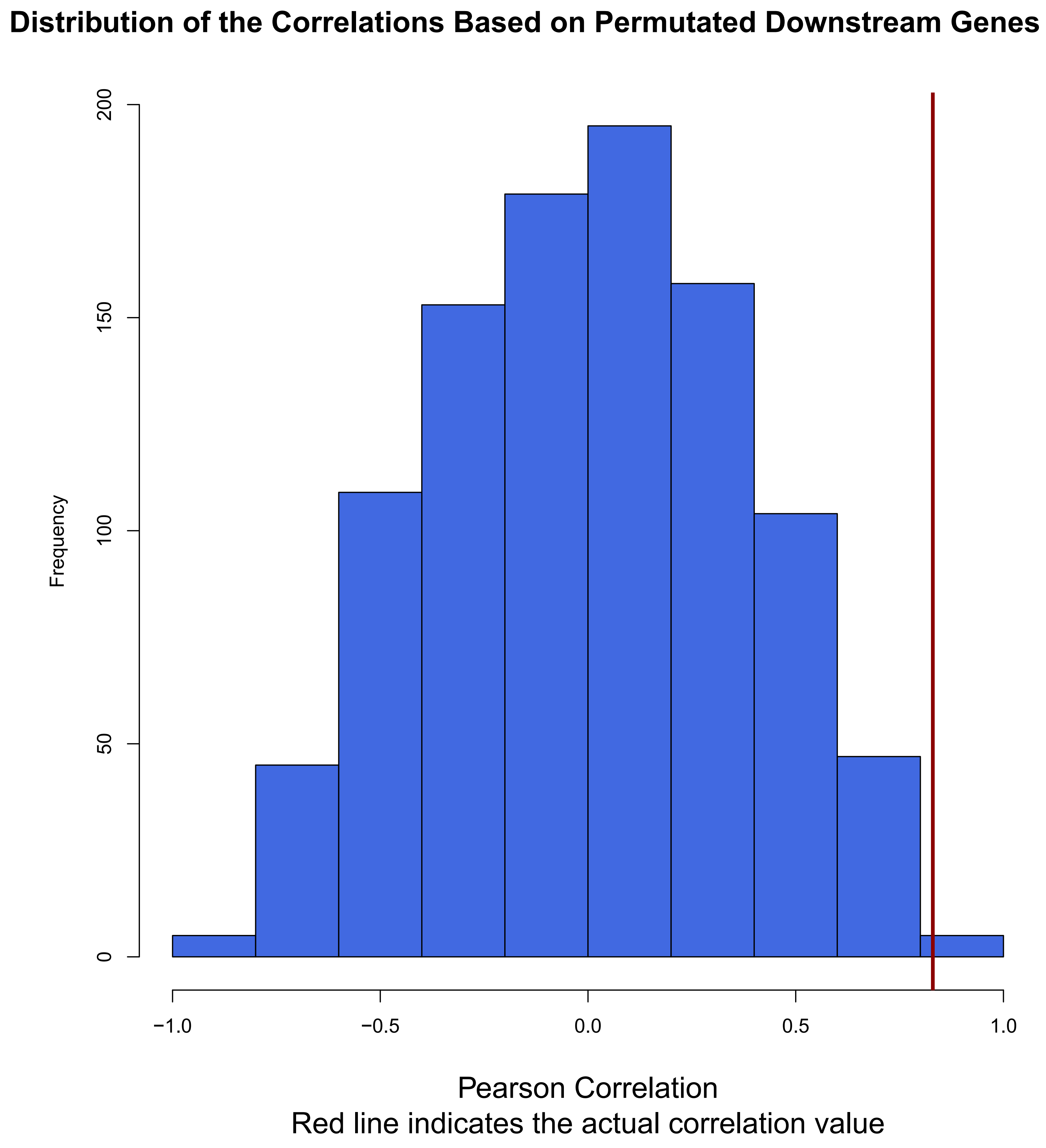
Distribution of the Correlations Based on Permutated Downstream Genes
Frequency
0
50
100
150
200
−1.0
−0.5
0.0
0.5
1.0
Pearson Correlation
Red line indicates the actual correlation value
